# Data on the effect of Cytopiloyne against *Listeria monocytogenes* infection in mice

**DOI:** 10.1016/j.dib.2016.03.044

**Published:** 2016-03-17

**Authors:** Chih-Yao Chung, Wen-Chin Yang, Chih-Lung Liang, Hsien-Yueh Liu, Shih-Kai Lai, Cicero Lee-Tian Chang

**Affiliations:** a Department of Veterinary Medicine, National Chung Hsing University, Taichung 402, Taiwan; b Agricultural Biotechnology Research Center, Academia Sinica, Taipei 11529, Taiwan; c Department of Aquaculture, National Taiwan Ocean University, Keelung 202, Taiwan; d Institute of Pharmacology, Yang-Ming University, Taipei 112, Taiwan; e Department of Life Sciences, National Chung-Hsing University, Taichung 402, Taiwan; f Department of Microbiology and Immunology, Institute of Microbiology and Immunology, Chung Shan Medical University, Taichung 402, Taiwan

## Abstract

Cytopiloyne (CP), a novel polyacetylene compound extracted from *B. pilosa*, shows a multi-bioactivity, including immunomodulatory and antidiabetes. Here, we investigated the anti-*Listeria* effect of cytopiloyne in mice by assessing mortality, clearance of *L. monocytogenes*, and pathology examination. The data presented herein are supplemental to our research article entitled “Cytopiloyne, a polyacetylenic glucoside from *Bidens pilosa*, acts as a novel anticandidial agent via regulation of macrophages” [1].

**Specifications Table**

**Table:** 

Subject area	*Biology*
More specific subject area	*Infection, immunity, and ethnopharmcology*
Type of data	*Figure, image*
How data was acquired	*Survival rate monitor, microscope, and serial dilution plate method*
Data format	*Analyzed*
Experimental factors	*Control and Cytopiloyne intraperitoneal injections at various concentration*
Experimental features	*Resistance of C57BL/6J mice to Listeria monocytogenes*
Data source location	*Taichung, Taiwan*
Data accessibility	*Data is within this article*

**Value of the data**

• Provides a mouse animal model for assessing the antimicrobial effect of herb compounds to intracellular pathogens.
• May be valuable for further studies to identify the molecular mechanism of CP involving in the intracellular survival of pathogens.
• May provide a new therapeutic strategy that uses a combination treatment of antimicrobial drugs and edible immunodulatory herbs to improve the efficacy of antimicrobial drug therapy on immunosuppressed patients.

## 1 Data

**Fig. 1:**
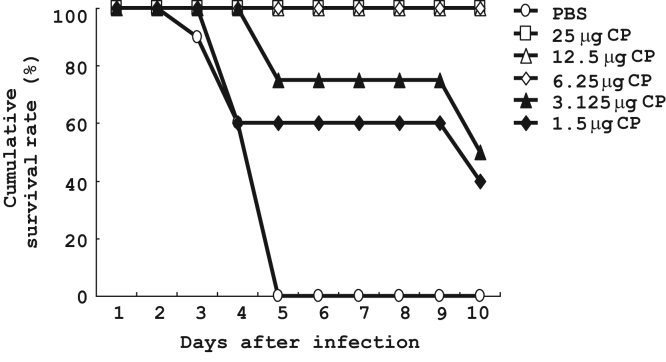
Cytopiloyne (CP) dose-dependently protected mice against *L. monocytogenes* infection (*n*=10 in each groups).

**Fig. 2:**
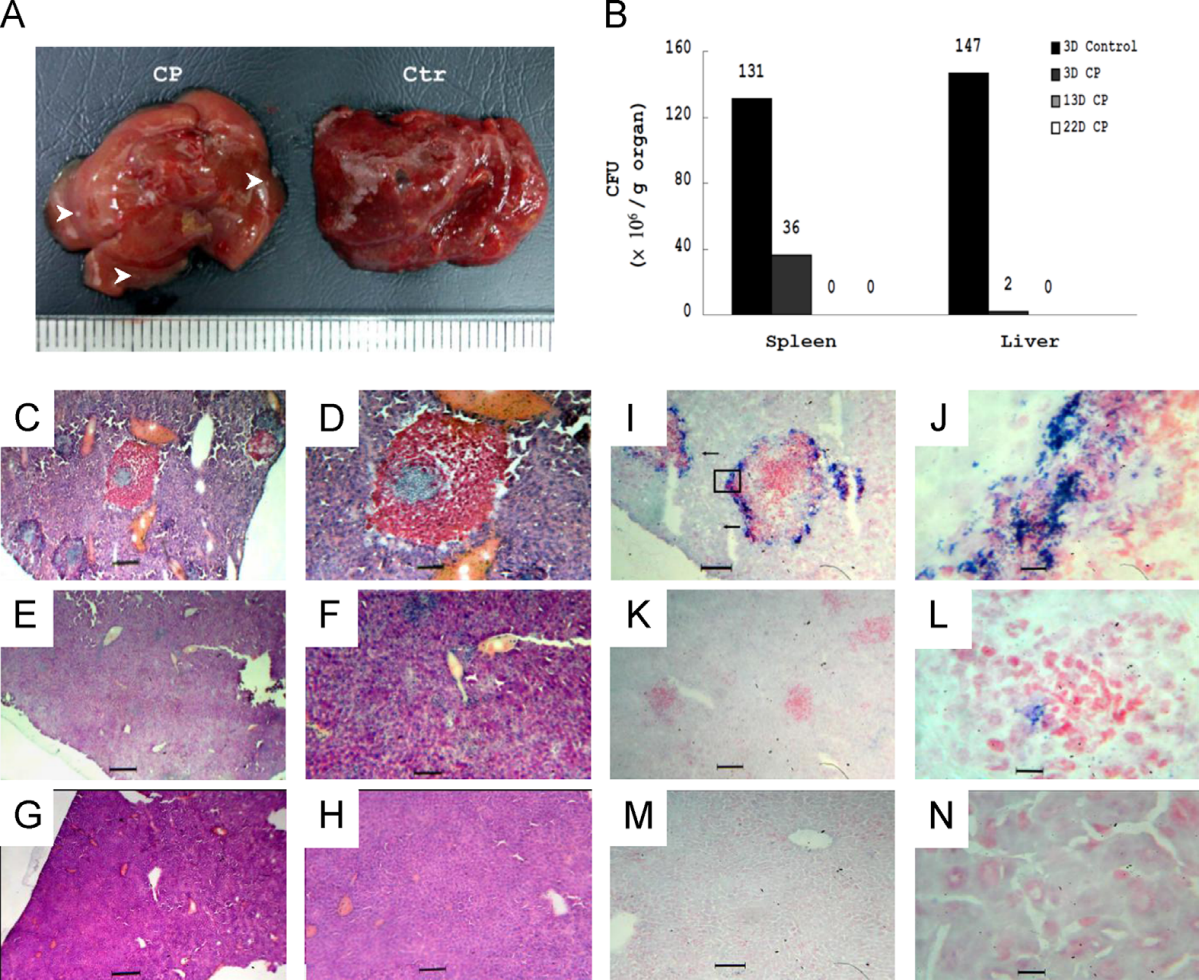
Cytopiloyne (CP) lowered the CFU counts and severity of lesions in *Listeria*-infection mice. (A) Gross pathology observation of livers of PBS-(Ctr) and CP-treated mice at 3 days post infection. Diffuse microabscesses were observed in liver of PBS-treated (Ctr) mice, while only focal microabscesses indicated by arrows were noted in CP-treated (CP) mice. (B) CP lowered the CFU counts in liver and spleen (*n*=3–6 in each groups). (C–H) Histopathology observation (H and E) of livers of PBS-(C and D, at 3 days post infection) and CP-treated (E and F, at 3 days post infection; G and H, at 13 days post infection) mice. C, E, and G: 100×; D, F, and H: 400×. (I–N) Histopathology observation (Gram staining) of livers of PBS-(I and J, at 3 days post infection) and CP-treated (K and L, at 3 days post infection; M and N, at 13 days post infection) mice. I, K, and M: 400×; J, L, and N: 1000×.

Here, we demonstrated that CP protected mice against *Listeria* infection by assessing the survival rate (*n*=10 in each groups; Fig. 1), gross pathology observation (Fig. 2A), the CFU counts in liver and spleen (Fig. 2B), and histopathology observations of frozen liver and spleen sections (Fig. 2C–N).

## 2 Experimental design, materials and methods

### 2.1 Chemicals, cells, and mice

*B. pilosa* plants were collected from Academia Sinica, Taiwan and authenticated by the Biodiversity Center of Academia Sinica. Cytopiloyne was prepared to 98% purity from whole plant of *B. pilosa* as previously described [2]. Briefly, Cytopiloyne was isolated on an RP-18 HPLC column by methanol extraction and ethyl acetate partition of whole *B. pilosa* plants. Structure and purity were confirmed by NMR spectra using a Bruker DMX-500 spectrometer and nuclear magnetic resonance determination, respectively. [2] *Listeria monocytogenes* (BCRC 15386) was obtained from Bioresource Collection and Research Center (Taiwan). Female 6–8 week-old C57BL/6J mice (National Laboratory Animal Center, Taiwan) were maintained and handled according to the guidelines of Academia Sinica Institutional Animal Care and Utilization Committee.

### 2.2 Listeria challenge

Six groups of 6 to 8-week-old C57BL/6J mice received intraperitoneal injection of PBS (control) or PBS solution of CP (1.5, 3.125, 6.25, 12.5 and 25 μg/kg BW) three times per week for 2 weeks. After 24 h, mice were intraperitoneally injected with *Listeria* (2×10^6^ CFU). The animals were then observed every day for determination of mortality [3].

### *2.3.* Clearance of *Listeria* and histopathology examination

2.3

As described in Section 2.2, C57BL/6 J mice received PBS (control) or PBS solution of CP (25 μg/kg BW) for 2 weeks and were intraperitoneally injected with *Listeria* (2×10^6^ CFU). The mice were then killed at 3, 13, and 22 days (if survival) post infection. Their liver and spleen were weighed and cut into 2 parts for CFU determination and histopathological examination. For CFU count, one part of the liver and spleen were homogenized, diluted and plated on BHI agar incubated at 37 °C for 12–16 h. The rest of the organs were frozen for tissue section and then stained by H&E and Gram staining (Hucker–Conn method).

## References

[1] C.Y. Chung, W.C. Yang, C.L. Liang, H.Y. Liu, S.K. Lai, C.L.T. Chang, Cytopiloyne, a polyacetylenic glucoside from Bidens pilosa, acts as a novel anticandidial agent via regulation of macrophages, J. Ethnopharmacol. 184 (2016) 72–80. http://ac.els-cdn.com/S0378874116300800/1-s2.0-S0378874116300800-main.pdf?_tid=ce994ce6-f244-11e5-a132-00000aacb360&acdnat=1458881484_034942659a73f7420a5953ade8122e0c 10.1016/j.jep.2016.02.036 26924565

[2] Chang C.L., Chang S.L., Lee Y.M., Chiang Y.M., Chuang D.Y., Kuo H.K. (2007). Cytopiloyne, a polyacetylenic glucoside, prevents type 1 diabetes in nonobese diabetic mice. J. Immunol. (Baltim., Md.: 1950).

[3] Chang S.L., Chiang Y.M., Chang C.L., Yeh H.H., Shyur L.F., Kuo Y.H. (2007). Flavonoids, centaurein and centaureidin, from Bidens pilosa, stimulate IFN-gamma expression. J. Ethnopharmacol..

